# Sialic acid catabolism drives intestinal inflammation and microbial dysbiosis in mice

**DOI:** 10.1038/ncomms9141

**Published:** 2015-08-25

**Authors:** Yen-Lin Huang, Christophe Chassard, Martin Hausmann, Mark von Itzstein, Thierry Hennet

**Affiliations:** 1 Institute of Physiology and Zurich Center of Integrative Human Physiology, University of Zurich, Zurich CH-8057, Switzerland; 2 Laboratory of Food Biotechnology, Institute of Food, Nutrition and Health, ETH Zurich, Zurich CH-8092, Switzerland; 3 Division of Gastroenterology and Hepatology, University Hospital of Zurich, Zurich CH-8006, Switzerland; 4 Institute for Glycomics, Griffith University, Gold Coast Campus, Gold Coast, Queensland 4222, Australia

## Abstract

Rapid shifts in microbial composition frequently occur during intestinal inflammation, but the mechanisms underlying such changes remain elusive. Here we demonstrate that an increased caecal sialidase activity is critical in conferring a growth advantage for some bacteria including *Escherichia coli (E. coli)* during intestinal inflammation in mice. This sialidase activity originates among others from *Bacteroides vulgatus*, whose intestinal levels expand after dextran sulphate sodium administration. Increased sialidase activity mediates the release of sialic acid from intestinal tissue, which promotes the outgrowth of *E. coli* during inflammation. The outburst of *E. coli* likely exacerbates the inflammatory response by stimulating the production of pro-inflammatory cytokines by intestinal dendritic cells. Oral administration of a sialidase inhibitor and low levels of intestinal α2,3-linked sialic acid decrease *E. coli* outgrowth and the severity of colitis in mice. Regulation of sialic acid catabolism opens new perspectives for the treatment of intestinal inflammation as manifested by *E. coli* dysbiosis.

## Abstract (web summary)

Intestinal inflammation is often associated with a shift in microbiota composition but the mechanisms are unclear. Here the authors show that an increase in caecal sialidase activity occurring during intestinal inflammation promotes the expansion of *Enterobacteriaceae*, which can lead to exacerbated inflammatory response.

## Graphical abstract

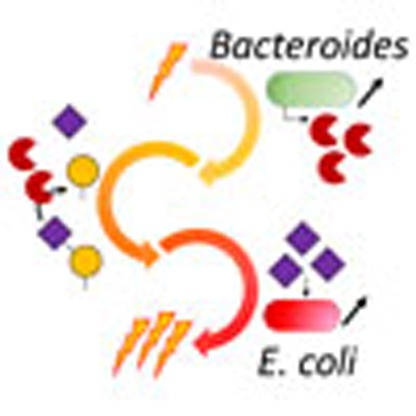

The intestinal microbiota has emerged as a key player in the regulation of physiological pathways and in the development of diseases. Along with intestinal diseases, such as necrotizing enterocolitis^1^ and inflammatory bowel disease^2^, gut microbiota contribute among others to the aetiology of diabetes^3^, asthma^4^, autoimmunity^5^ and cancer^6^. Accordingly, much effort has been dedicated in understanding the factors influencing the composition of the intestinal microbiota to maintain or restore health in the host organism.

Carbohydrates are a major class of food products that profoundly affect the gut microbiota. Whereas most monosaccharides are absorbed by the small intestine, oligo- and polysaccharides are not digested in the upper gastrointestinal tract and reach the colon intact. The impact of complex carbohydrates on microbial composition is based on the expression of specific hydrolases^7^, which enable some bacterial species to process and utilize breakdown products as nutrients, thereby conferring a proliferative advantage over bacteria that cannot process complex carbohydrates^8^. The first carbohydrates ingested just after birth are provided by breast milk, which is a rich source of lactose and oligosaccharides^9^. The uptake of milk oligosaccharides coincides with the microbial colonization of the gut and favors the proliferation of bacteria equipped with carbohydrate-processing enzymes, such as *Bifidobacterium* and *Bacteroides* spp. that are enriched in breast-fed infants^10^.

In addition to food carbohydrates, several intestinal bacteria can process host-derived carbohydrates, which are prominent constituents of mucosal layers. Besides providing carbon sources for bacterial growth, released host-derived carbohydrates influence gene expression in the microbiota, thereby affecting the virulence of pathogenic bacteria as demonstrated by the regulation of virulence factors in enterohaemorrhagic *Escherichia coli* by fucose^11^. Other host-derived carbohydrates, such as sialic acids, are taken up by bacteria lacking *de novo* biosynthetic pathways for these sugars, and incorporated into bacterial capsule and lipooligosaccharides^12^. The decoration of bacterial glycoconjugates with sialic acid protects microbes from recognition by the host immune system^13^ and regulates the host immune response through interactions with sialic acid-binding lectins^14^. Finally, the interplay between intestinal microbiota and host glycosylation is not limited to the utilization of host glycans by bacteria. Sialic acids as terminal residues on intestinal glycoconjugates are a prime target for bacterial adhesins and toxins from *Vibrio cholerae*, *Helicobacter pylori* and *E. coli*^15^,^16^.

The structural complexity of carbohydrates, either ingested in the form of milk oligosaccharides or expressed as host-derived glycans, hampers the elucidation of their impact on the gut microbiota. Accordingly, little is known about the relevance of specific carbohydrates on microbiota composition and on intestinal physiology. The application of mice deficient for glycosyltransferases enables the investigation of interactions between defined carbohydrates, intestinal microbes and the host immunity. For example, a study of α1,2-fucosyltransferase *Fut2* knockout mice has recently demonstrated the interplay between fucosylated glycans and diet polysaccharides on shaping the gut microbiota^17^. The study of α2,3 sialyltransferase *St3gal4* knockout (ST) mice, which mediates α2,3-sialyllactose (3SL) synthesis in mammary gland, has established the role of the milk oligosaccharide on the gut microbiota and thereby on the susceptibility of mice in dextran sulphate sodium (DSS)-induced acute^18^ and chronic colitis^19^. Through the investigation of DSS-mediated colitis in ST mice and the modulation of the intestinal microbiota by selective antibiotic treatment, the present study reveals the critical role of α2,3-linked sialic acid in establishing a niche for intestinal *E. coli* after lactation and during intestinal inflammation.

## Results

### Gut microbiota change during DSS-induced colitis

**Figure 1:**
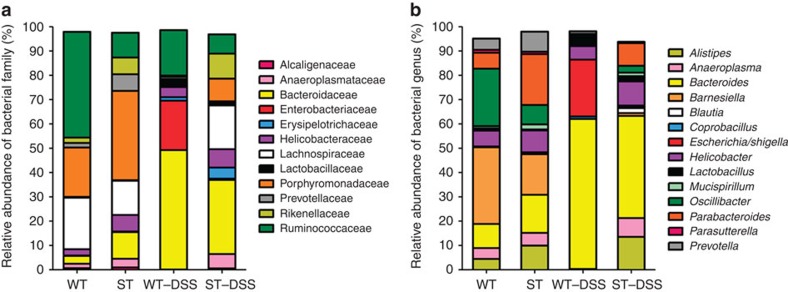
Bacterial composition in mice with DSS-induced colitis. (**a**) 16S rRNA pyrosequencing analysis of faecal microbial taxa families in control and DSS-treated WT and ST mice (at day 8 after DSS addition). (**b**) Pyrosequencing analysis of faecal microbial taxa at the genus level. Data show the average percentage of total identified sequences obtained from a pool of eight mice per group. Only the bacterial taxa representing at least 1% of total identified sequences are presented.

To unravel the relationship between α2,3-linked sialic acid and the intestinal microbiota, and to identify the mechanisms of α2,3-linked sialic acid effects on colitis development, we have first addressed the impact of intestinal bacterial groups on colitis by treating mice with a panel of antibiotics. Correlations between the resulting changes in microbial composition and susceptibility to DSS-mediated colitis pointed to specific bacterial families possibly regulating the severity of colitis in wild-type (WT) and ST mice. In fact, the composition of colonic bacteria in WT and ST mice differed at the adult stage. The most abundant bacterial family in WT mice was *Ruminococcaceae*, reaching 44% of total bacteria. By contrast, *Ruminococcaceae* only represented 10% of colonic bacteria in ST mice, whereas *Porphyromonadaceae* dominated by reaching 37% (Fig. 1a). The bacterial composition of mice undergoing intestinal inflammation induced by DSS changed markedly, as seen by a strong expansion of *Bacteroidaceae* and *Enterobacteriaceae* in WT mice. ST mice, which were less susceptible to DSS than WT mice^18^, also showed increased *Bacteroidaceae* levels during DSS challenge, whereas *Enterobacteriaceae* remained at low level. Sequence analysis at the genus level indicated that the *Escherichia* and *Shigella* accounted for the observed increase of *Enterobacteriaceae* in WT mice, and *Bacteroides* accounted for the increase of *Bacteroidaceae* in both WT and ST mice under DSS challenge (Fig. 1b).

### Antibiotics effect on DSS-induced colitis

**Figure 2:**
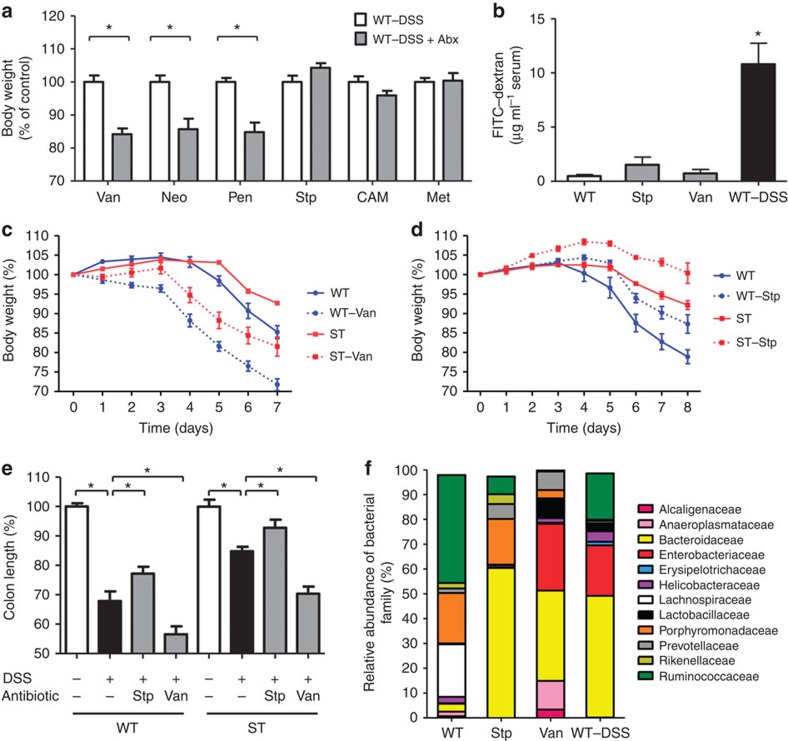
Antibiotics effect on DSS-induced colitis. (**a**) Mice were treated with vancomycin (Van), neomycin (Neo), penicillin (Pen), streptomycin (Stp), chloramphenicol (CAM) and metronidazole (Met) for 3 weeks before DSS challenge for 5 days. Body weight was measured by day 8 after initiation of DSS challenge and given as percentage to the body weight of mice challenged with DSS without antibiotics. The data are represented as mean±s.e.m. *N*=6–8, *P<0.05 (two-tailed Student's *t*-test). (**b**) Intestinal permeability was measured by FITC–dextran levels in the serum from control, colitogenic mice on day 5 of DSS challenge, and 3 weeks of antibiotic pretreated mice. *N*=5, *P<0.05 (ANOVA, Bonferroni's multiple comparison test). (**c**) Relative change in body weight of WT and ST mice treated with 0.5 g l^−1^ Van for 7 days and 3% DSS for 5 days; control mice received DSS without Van. (**d**) Relative change in body weight of WT and ST mice treated with 1 g l^−1^ Stp for 8 days and 3% DSS for 5 days. (**e**) Colon length was determined at the end point of DSS treatment. In (**c**–**e**) the data are represented as mean±s.e.m. from two independent experiments, *N*=6-8, *P<0.05 (ANOVA, Bonferroni's multiple comparison test). (**f**) 16S rRNA pyrosequencing analysis of faecal microbial taxa families in untreated WT mice, in Van, Stp-treated WT mice and DSS-challenged WT mice (WT–DSS). The data are represented as the percentage of total identified sequences obtained from a pool of eight mice per group.

To determine whether a specific group of bacteria accounted for the different response to DSS, we treated WT mice with a panel of antibiotics before DSS challenge. Vancomycin, neomycin and penicillin exacerbated the severity of DSS-induced colitis as monitored by loss of body weight (Fig. 2a). Streptomycin was the only antibiotic that attenuated the loss of body weight during colitis, whereas chloramphenicol and metronidazole did not have much impact on the course of the inflammatory response (Fig. 2a). To exclude any damaging effect caused by the use of antibiotics on intestinal barrier function, we assessed epithelial permeability by measuring the leakage of orally administered fluorescein isothiocyanate (FITC)–dextran into the bloodstream. Permeability was only significantly increased after DSS ingestion, but not after antibiotic treatment (Fig. 2b). We also tested the effect of a short-term treatment with antibiotics to exclude possible adaptations of the host mucosa to three weeks of altered microbiota composition. Focusing on vancomycin (Fig. 2c) and streptomycin (Fig. 2d), we could reproduce the worsening and improving effects of these two antibiotics by only administering vancomycin and streptomycin during DSS challenge. The protective effect of streptomycin towards DSS challenge was even more pronounced in a short-term treatment compared with a 3-week pre-treatment in both WT and ST mice. The extent of intestinal inflammation was confirmed by measuring the length of the colon in treated mice. The shortening of colon length induced by DSS ingestion was aggravated by vancomycin and reduced by streptomycin (Fig. 2e). The impact of vancomycin and streptomycin treatment on the intestinal microbiota of WT mice was analysed by 16S rRNA pyrosequencing and compared with the changes observed during DSS challenge. Vancomycin induced a strong increase of *Enterobacteriaceae*, which raised to 27% of total bacteria, whereas *Enterobacteriaceae* remained below 0.1% of total bacteria in streptomycin-treated mice (Fig. 2f). Under both antibiotics, *Bacteroidaceae* expanded to represent the major bacterial family, but the increase in *Bacteroidaceae* did not directly correlate with the severity of DSS-induced colitis. In contrast, the abundance of *Enterobacteriaceae* correlated with the magnitude of colitis. Overgrowth of *Enterobacteriaceae* and several *Bacteroidaceae* spp. during intestinal inflammation is well documented, although the mechanisms underlying such expansions have not been identified in previous studies^20^,^21^.

### Expansion of *E. coli* during DSS-induced colitis

**Figure 3:**
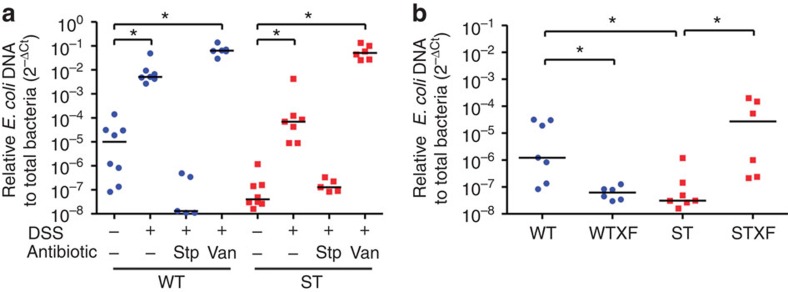
Intestinal *E. coli* in mice. Relative abundance of *E. coli* was determined by real-time PCR using specific *uidA* primers and quantified using the 2^−▵Ct^ method. (**a**) Relative abundance of *E. coli* in groups of 6-week-old WT and ST mice treated with the antibiotics vancomycin (Van) and streptomycin (Stp) and challenged with 3% DSS. Mice were treated for 8 days with or without antibiotics and DSS for the first 5 days in drinking water. Control mice received sterile drinking water. (**b**) Relative abundance of *E. coli* in 6-week-old WT, ST and respectively cross-fostered (XF) mice, which were fed by foster mothers of the other genotype during lactation. The data are represented as median values. Each point indicates a single mouse from two independent experiments. *N*=6–8, *P<0.05 (ANOVA, Bonferroni's multiple comparison test).

To verify which species of *Enterobacteriaceae* expanded during intestinal inflammation, we applied specific primers targeting the β-glucuronidase *uidA* gene^22^ and confirmed a significant increase of *E. coli* in DSS-challenged mice and in vancomycin-treated mice (Fig. 3a). In contrast, a significant reduction of *E. coli* was observed in both WT and ST mice treated with streptomycin. Overall, *E. coli* levels correlated with the severity of colitis in all genotypes and antibiotic treatments tested. Of note, the level of *E. coli* in adult ST mice was 2 orders of magnitude lower than in WT mice, indicating that decreased exposure to α2,3-linked sialic acid, because of reduced sialylation of host glycans and the absence of 3SL in milk ingested during lactation, was accompanied by low level of intestinal *E. coli*. The importance of milk 3SL during lactation at promoting the low-level colonization of *E. coli* was also visible in WT mice that were fostered by ST mother during lactation. WT mice fed on 3SL-deficient milk (WTXF) showed lower *E. coli* levels than littermates fed on normal milk. Similarly, ST mice fed on normal milk showed elevated *E. coli* levels compared with littermates fed on 3SL-deficient milk (Fig. 3b). The relative abundance of intestinal *E. coli* in cross-fostered mice also reflected the severity of DSS-induced colitis^18^. Overall, these data confirmed that neonatal exposure to milk 3SL contributed to establishing an intestinal niche for *E. coli*, thereby providing a ground for the subsequent expansion of *E. coli* during colitis induced by DSS.

### Exposure to sialic acid promotes *E. coli* expansion

**Figure 4:**
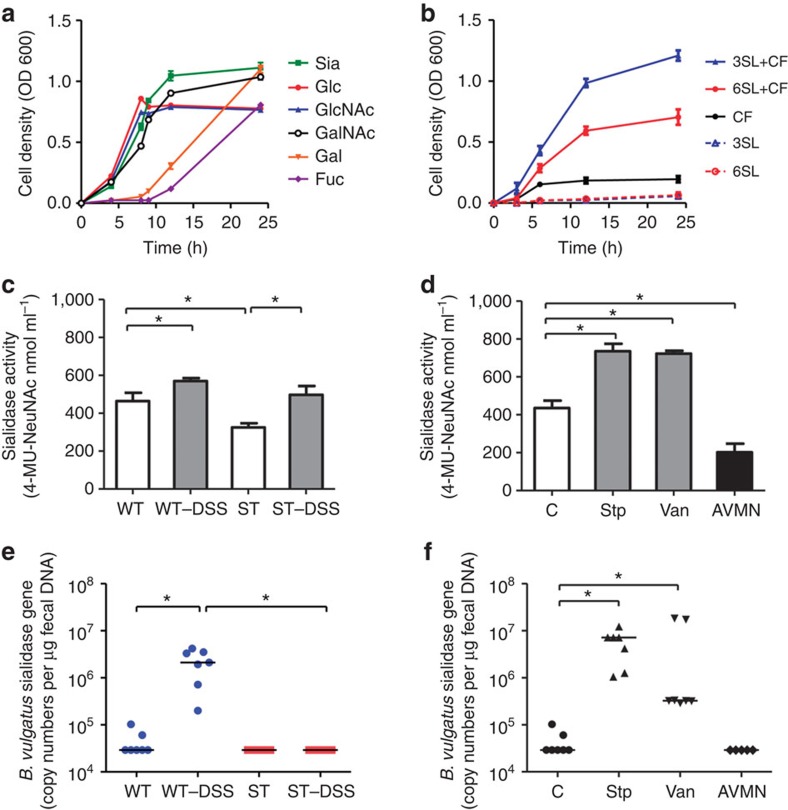
Sialic acid processing and uptake by *E. coli in vitro*. (**a**) Growth of *E. coli* EHV2 in M9 minimal medium containing single monosaccharide at 10 mM. Sia, *N*-acetylneuraminic acid; Glc, glucose; GlcNAc, *N*-acetylglucosamine; GalNAc, *N*-acetylgalactosamine; Gal, galactose; Fuc, fucose. (**b**) Growth of *E. coli* in minimal medium containing 5 mM of 3SL and 6SL with and without supplementation of caecal fluid (CF, 1.5%, v/v) derived from WT mice. (**c**) CF of conventional and DSS-challenged WT and ST mice were collected. Sialidase activity was determined by measuring fluorescent 4-MU-NeuNAc at 440 nm. (**d**) Sialidase activity was also measured in the CF of WT and WT mice treated with either streptomycin (Stp 1 g l^−1^), vancomycin (Van 0.5 g l^−1^) or antibiotic cocktail (AVMN: ampicillin, Van, metronidazole and neomycin). In (**c**–**d**), the data are represented as mean±s.e.m. from two independent experiments, *N*=5–8, *P<0.05 (two-tailed Student's *t*-test). (**e**) The abundance of the *B. vulgatus* BVU_4143 sialidase gene was determined by real-time PCR from caecum samples of WT and ST mice, and (**f**) caecum samples of antibiotic-treated WT mice. The data are represented as gene copy number per μg of faecal DNA. In (**e**–**f**) each data point indicates a single mouse from two independent experiments, *N*=5–7, *P<0.05 (two-tailed Student's *t*-test).

To address the impact of 3SL on *E. coli* proliferation, we isolated various strains of commensal *E. coli* from the colon of WT mice during DSS-induced colitis. The identity of the isolated bacteria with *E. coli* was confirmed by sequencing universal stress protein *uspA* gene^23^, gyrase *gyrB* gene^24^ and by biochemical testing using the API-20E *Enterobacteriaceae* detection system. The culture of the isolated *E. coli* strain EHV2 in minimal medium containing unique monosaccharides as carbon source confirmed that *N*-acetylneuraminic acid was a preferential source of energy for *E. coli* (Fig. 4a). In contrast to free *N*-acetylneuraminic acid, the milk oligosaccharides 3SL and α2,6-sialyllactose (6SL) did not support *E. coli* growth *in vitro* (Fig. 4b). Proliferation could, however, be restored by adding sterile-filtered caecal fluid from WT mice to the culture medium, whereas *E. coli* growth was more robust in 3SL than in 6SL containing minimal medium. Because *E. coli* do not produce sialidases, the restoration of bacterial growth pointed to the presence of an α2,3-preferential sialidase activity in caecal fluid. Such a sialidase activity was confirmed in caecal fluid and shown to increase significantly during DSS-induced colitis in both WT and ST mice (Fig. 4c). The substrate specificity of this caecal fluid sialidase was demonstrated by high-performance liquid chromatography (HPLC) analysis after incubation of 3SL with caecal fluid (Supplementary Fig. 1). The sialidase activity was also increased in the caecal fluid of WT mice treated with streptomycin or vancomycin. By contrast, the sialidase activity was strongly decreased in WT mice treated with a broad-spectrum antibiotic cocktail consisting of ampicillin, vancomycin, metronidazole and neomycin, which supported the bacterial origin of the sialidase activity in caecal fluid (Fig. 4d). To identify the source of this sialidase activity, we focused on bacterial groups, such as commensal *Bacteroides* and *Bifidobacteria* species that are known to secrete sialidases^25^. Host-derived sialidases were unlikely candidates since vertebrate sialidases are unstable as soluble proteins in the extracellular space^26^.

### *Bacteroides vulgatus* sialidase releases sialic acid

As *Bacteroides* species were strongly increased in the gut of mice challenged with DSS (Fig. 1b) as well as in mice treated with streptomycin or vancomycin (Fig. 2f), we searched for sialidase genes in the caecum of DSS-challenged mice using a series of PCR primers encompassing known *Bacteroides* sialidase sequences in the glycoside hydrolase family 33 of the CAZy database. This analysis revealed a 100-fold increase in copy number of the *B. vulgatus* BVU_4143 sialidase gene (gene ID: 5305102) in WT mice treated with DSS, whereas no change was detected in ST mice (Fig. 4e). The low levels of BVU_4143 sialidase gene in ST mice, however, shows that *B. vulgatus* is not the only source of sialidase activity in these mice. The abundance of *B. vulgatus* also increased accordingly in the colon of WT mice during DSS-induced colitis (Supplementary Fig. 2). Moreover, the abundance of the BVU_4143 sialidase gene increased in WT mice treated with streptomycin and vancomycin, but decreased in mice treated with antibiotic cocktail (Fig. 4f).

The sialidase activity of BVU_4143 was confirmed after expression as a recombinant protein by demonstrating its ability to hydrolyse the aryl substrate 4-methylumbelliferyl *N*-acetylneuraminic acid (Supplementary Fig. 3a) and 2-O-(4-Nitrophenyl) *N*-acetylneuraminic acid (Supplementary Fig. 3b). The sialidase activity of recombinant BVU_4143 was inhibited by the sialidase inhibitor *N*-acetyl-2,3-didehydro-2-deoxyneuraminic acid (Neu5Ac2en) and lost by heat treatment. The addition of recombinant BVU_4143 to minimal media containing 3SL or 6SL enabled the growth of *E. coli* EHV2 on these oligosaccharides (Supplementary Fig. 3c), as observed for the restoration of *E. coli* EHV2 proliferation by addition of sterile-filtered caecal fluid to culture medium containing 3SL and 6SL (Fig. 4b). Overall, these results indicate that the expansion of *B. vulgatus* and the concomitant increased sialidase activity during DSS-induced colitis enables the sialic acid-dependent outgrowth of *E. coli* during inflammation.

### Sialic acid is required for sustaining *E. coli* colonization

**Figure 5:**
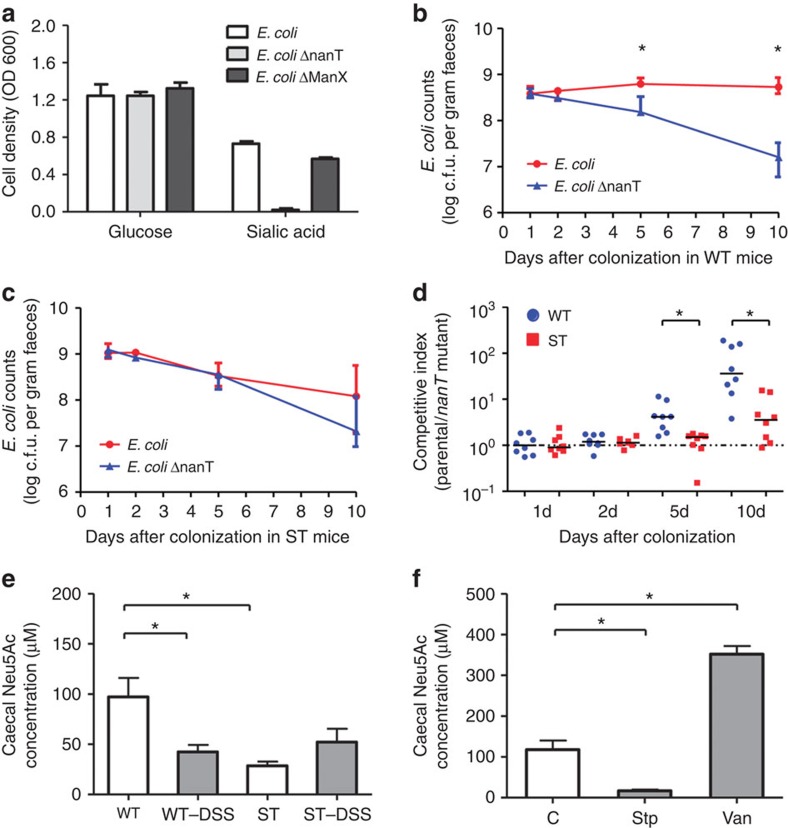
Sialic acid catabolism is required for maintaining *E. coli* colonization. (**a**) Growth of parental *E. coli*, *nanT* mutant and *ManX* mutant in modified minimal media containing 10 mM of glucose or sialic acid. (**b**) Relative fitness of parental *E. coli* and *nanT* mutant in colonization of WT mice and (**c**) ST mice. Bacterial colonization fitness was determined by counting c.f.u. of serially diluted faecal faeces collected at indicated time points. In (**b**,**c**), data are shown as median±interquartile range. *N*=8, *P<0.05 (two-tailed Student's *t*-test). (**d**) Competitive index was calculated as ratio of parental *E. coli* to *nanT* mutant in WT and ST mice over 10 days after administration. The data are represented as median value, and each dot indicates an individual animal. *N*=8, *P<0.05 (two-tailed Student's *t*-test). (**e**) Levels of free Neu5Ac in the caecal fluid of control and DSS-challenged WT mice were determined by HPLC analysis. (**f**) Levels of free Neu5Ac in the caecal fluid of control (C), streptomycin- and vancomycin-treated mice measured 5 days post treatment. In (**e**,**f**), the data are represented as mean±s.e.m. from two independent experiments. *N*=6–8, *P<0.05 (ANOVA, Bonferroni's multiple comparison test).

The dependence of *E. coli* on sialic acid *in vivo* was investigated by deleting the sialic acid transporter *nanT* gene^27^. Disruption of *nanT*, the first committed step in the sialic acid utilization pathway, abolished growth of the mutant *E. coli* strain in a sialic acid-containing minimal medium, but not growth in glucose-containing medium (Fig. 5a). By contrast, the disruption of mannose transporter *ManX* did not affect the growth of *E. coli* in both glucose and sialic acid-containing medium. To investigate the *in vivo* colonization efficiency, *nanT* mutant and parental *E. coli* were gavaged at equal amounts of each 10^6^ cells to ampicillin-pretreated mice. Colonization efficiency was determined over a period of 10 days after inoculation by counting *E. coli* isolated from freshly isolated faeces samples. Both strains were colonized at 10^8^–10^9^ colony-forming units (c.f.u.) per gram faeces in WT mice by 2 days after inoculation. Parental *E. coli* remained stable over 10 days, but *nanT* mutants decreased markedly over the same period (Fig. 5b). The same experiment performed in ST mice showed that even parental *E. coli* did not maintain their original levels in an environment deficient of α2,3-linked sialic acid (Fig. 5c). The comparison of competitive index between parental and *nanT E. coli* in the intestines of WT and ST mice suggested that the growth advantage of parental *E. coli* was associated with the local availability of sialic acid (Fig. 5d). The levels of free Neu5Ac in the caecal fluid were indeed higher in WT mice than in ST mice (Fig. 5e), thereby correlating with the occurrence of intestinal *E. coli* in WT and ST mice (Fig. 3a). The concentrations of Neu5Ac measured in the caecum of WT mice treated with streptomycin and vancomycin (Fig. 5f) also matched the abundance of *E. coli* in these mice (Fig. 3a), but the low levels of Neu5Ac in streptomycin-treated mice also indicated that other bacteria consumed this carbohydrate when *E. coli* was suppressed. In fact streptomycin treatment increased the abundance of *Bacteroidaceae* and *Porphyromonadaceae* (Fig. 2f) that include several sialidase producers and sialic acid consumers, such as *Bacteroides fragilis* and *Parabacteroides distasonis*. In the late stage of DSS-induced colitis, the levels of free Neu5Ac decreased in WT mice and relatively increased in ST mice, which reflected increased sialic acid usage by *E. coli* and increased sialidase activity (Fig. 4c) during intestinal inflammation. Accordingly, these results were consistent with the hypothesis that *E. coli* outgrowth in the intestine depends on the release of sialic acid from host glycans.

### Sialidase inhibition lowers *E. coli* expansion and colitis

**Figure 6:**
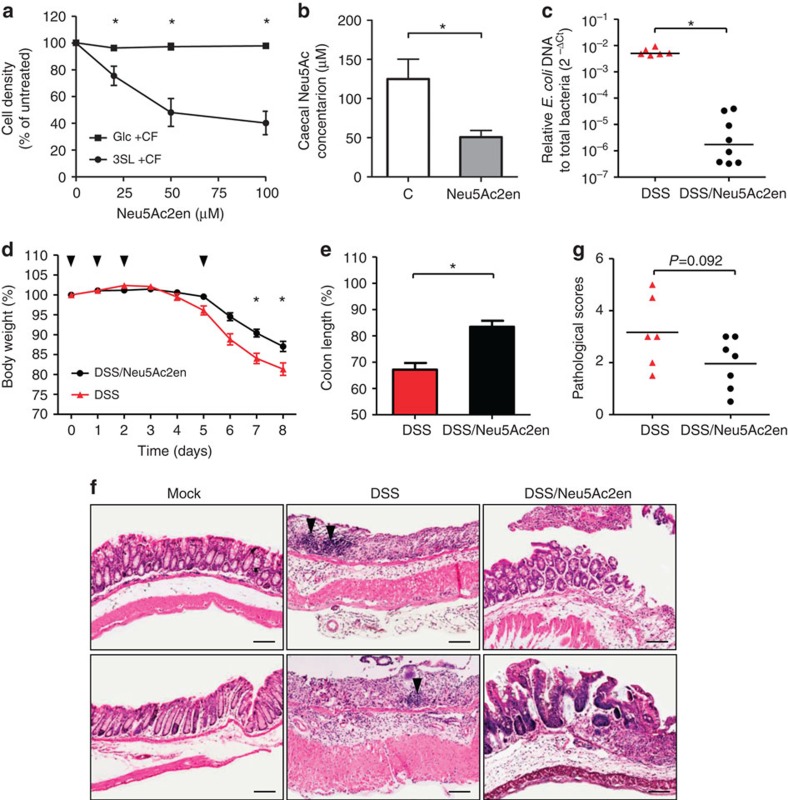
Reduced intestinal *E. coli* expansion by sialidase inhibition. (**a**) *In vitro* growth of *E. coli* EHV2 in minimal media containing 10 mM Glc or 3SL and the presence of caecal fluid and inhibitor Neu5Ac2en (0, 20, 50 and 100 μM). (**b**) Levels of Neu5Ac in the caecal fluid of control, and inhibitor-treated WT mice (2 h post 0.5 mg Neu5Ac2en administration) were measured by HPLC analysis. Control mice were administered with sterile PBS. In (**a**,**b**), the data are represented as mean±s.e.m. from two independent experiments, *N*=5, *P<0.05 (two-tailed Student's *t*-test). (**c**) Relative abundance of intestinal *E. coli* in control (PBS-treated) and Neu5Ac2en-treated WT mice (10 mg kg^−1^ per day) was determined at day 8 after initiation of DSS challenge. Data are represented as median values from two independent experiments, *N*=6–8, *P<0.05 (two-tailed Student's *t*-test). (**d**) Relative change in body weight of control and Neu5Ac2en-treated WT mice during DSS challenge for 5 days. Arrowheads indicate the time points of Neu5Ac2en administration. (**e**) Colon length was measured at day 8 after initiation of DSS challenge. In (**d**,**e**), the data are represented as mean±s.e.m. from two independent experiments, *N*=6–8, *P<0.05 (two-tailed Student's *t*-test). (**f**) Representative histological sections of colon tissues from untreated WT mice (Mock), DSS-treated mice (DSS) and DSS-treated mice administered with sialidase inhibitor (DSS/Neu5Ac2en). Arrowheads indicate infiltrating leukocytes. Scale bar, 100 μm. (**g**) Scoring of colitis severity by quantitative examination of tissue alteration and leukocytes infiltration. Each dot indicates an individual animal from two independent experiments.

On the basis of the requirement for sialidase activity to cleave α2,3-linked sialic acid and to promote *E. coli* proliferation during intestinal inflammation, we hypothesized that sialidase inhibition would decrease both the expansion of *E. coli* during DSS-induced colitis and the severity of the inflammatory response. We first confirmed the effectiveness of the sialidase inhibitor Neu5Ac2en at preventing *E. coli* growth in presence of 3SL and caecal fluid *in vitro* (Fig. 6a). Next, we confirmed the effectiveness of Neu5Ac2en at reducing the release of sialic acid *in vivo* by showing decreased levels of free Neu5Ac in the caecum of WT mice treated with the sialidase inhibitor (Fig. 6b). Oral administration of Neu5Ac2en to WT mice during DSS challenge also prevented the outgrowth of *E. coli* during inflammation, as seen by a decrease of *E. coli* levels by 2–3 orders of magnitude (Fig. 6c), and decreased the severity of DSS-induced colitis as assessed by change in body weight (Fig. 6d) and colon length (Fig. 6e). Neu5Ac2en treatment also reduced the loss of colonic architecture and leukocyte infiltration (Fig. 6f), although without reaching statistical significance (Fig. 6g). Neu5Ac2en treatment was by contrast ineffective in ST mice challenged with DSS (Supplementary Fig. 4), which was expected considering the low levels of sialidase-producing *Bacteroides* spp. and low sialidase activity in the caecum of ST mice. Overall, these data demonstrated that inhibition of caecal sialidase activity significantly reduced the outburst of *E. coli* and hence the severity of colitis in mice.

### *E. coli* intensifies dendritic cell activation

**Figure 7:**
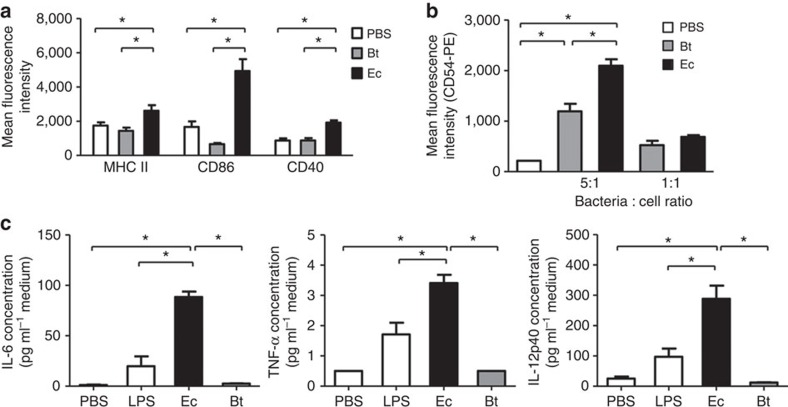
Stimulation of DCs and monocytes. (**a**) In stimulation assay, bacteria were fixed with 0.5% paraformaldehyde in PBS, washed and co-cultured at a ratio of 100:1 with mouse mesenteric CD11c^+^ DCs. Ec, *E. coli* EHV2; Bt, *B. thetaiotaomicron*. Cell surface expression of CD86, CD40 and MHC-II was analysed by flow cytometry. (**b**) Human monocytic THP-1 cells were stimulated with fixed bacteria at ratios of 5:1 and 1:1 to cells for 14 h at 37 °C. Cell surface expression of CD54 was analysed by flow cytometry. In (**a**,**b**), data are shown as mean fluorescence intensity, MFI±s.e.m. from three independent experiments. *N*=6, *P<0.05 (ANOVA, Bonferroni's multiple comparison test). (**c**) Cytokine expression in the culture supernatant of stimulated mesenteric CD11c^+^ DCs stimulated for 14 h at 37 °C with fixed Ec, Bt and 500 ng ml^−1^ of LPS. PBS was used as negative stimulation control. Data are shown as mean±s.e.m. from two independent experiments. *N*=4–5, *P<0.05 (ANOVA, Bonferroni's multiple comparison test).

The question as to how *E. coli* proliferation affected intestinal inflammation remained open. We therefore assessed the pro-inflammatory potential of *E. coli* on intestinal CD11c^+^ dendritic cells (DCs). Previous work has shown increased CD11c^+^ DC infiltration to the colonic mucosa of WT mice compared with ST mice, suggesting a critical role of DCs during intestinal inflammation^19^. We examined the stimulatory effect of *E. coli* EHV2 and of the *Bacteroides thetaiotaomicron* on mesenteric lymph node-derived CD11c^+^ DCs. *B. thetaiotaomicron* was chosen as reference because *Bacteroides* represents a major group of intestinal bacteria, which expanded in both WT and ST mice during DSS-mediated colitis (Fig. 1b). Stimulation of CD11c^+^ DCs with fixed *E. coli* increased the expression of the activation markers major histocompatibility complex (MHC)-II, CD86 and CD40, whereas stimulation with fixed *B. thetaiotaomicron* failed to activate CD11c^+^ DCs (Fig. 7a). The pro-inflammatory effect of *E. coli* was not limited to mouse DCs as stimulation of the human monocytic cell line THP-1 also increased the expression of the activation marker CD54 (Fig. 7b). The effect of *E. coli* was even more pronounced when measuring the secretion of pro-inflammatory cytokines from stimulated mouse CD11c^+^ DCs. The levels of interleukin (IL)-6, tumour-necrosis factor (TNF)-α and IL-12p40 produced after *E. coli* stimulation exceeded those reached after stimulation with lipopolysaccharides (LPSs) at 500 ng ml^−1^. Under identical conditions, *B. thetaiotaomicron* did not increase cytokines production (Fig. 7c).

Overall, this study demonstrated that the expansion of commensal *E. coli* following the alteration of epithelial integrity caused by DSS uptake was mediated by increased exposure to sialic acid, and that overgrowth of *E. coli* exacerbated intestinal inflammation by stimulating the release of pro-inflammatory cytokines from intestinal DCs.

## Discussion

Multiple studies have documented that intestinal inflammation is frequently accompanied by imbalanced microbiota. Such a dysbiosis is often characterized by a relative increase of facultative anaerobic *Enterobacteriaceae*^28^. Different factors such as nitrate^29^ and enterobactin^30^ promote *Enterobacteriaceae* expansion. The present study underlines the contribution of host glycosylation, specifically of α2,3-linked sialic acids, in enabling the proliferation of *Enterobacteriaceae* during intestinal inflammation in mice. Exposure to α2,3-linked sialic acids begins during lactation with the uptake of the milk oligosaccharide 3SL. After weaning, sialylated host glycans constitute the main source for the carbohydrate. Whereas, *Enterobacteriaceae* genomes encode various glycosidases, bacteria such as *E. coli* cannot digest sialylated oligosaccharides. Therefore, their growth relies on scavenging free monosaccharides released by glycosidases of other bacteria. Our comparative study of monosaccharides showed that the sialic acid yielded the fastest growth of *E. coli* among the main monosaccharides encountered in mammalian glycans. This finding is consistent with previous work showing that sialic acid catabolism conferred a growth advantage to intestinal *E. coli*^31^,^32^.

The growth advantage provided by sialic acid was dependent on a sialidase displaying a preference for α2,3-linked over α2,6-linked sialic acids, and which increased during intestinal inflammation. Commensal *E. coli* do not express sialidases to liberate host sialylated glycans, therefore the access to bound sialic acids depends on secreted sialidases, such as the BVU_4143 sialidase identified in our study. We also detected other sialidase genes in colitogenic mice, such as sequences sharing similarity with sialidase genes encoded by *B. fragilis* and *P. distasonis*, although the abundance of these sialidase sequences did not vary between mouse genotypes and during DSS-mediated colitis. The dependence of *E. coli* on sialidases secreted by *Bacteroides* spp. may explain the parallel increased abundance of *Bacteroides* spp. and *E. coli* observed in patients with colitis^33^. A recent study also demonstrated that the commensal sialidase-producing *B. thetaiotaomicron* was associated with proliferation of *Salmonella enterica* typhimurium and *Clostridium difficile*^34^. By contrast, colonization of mice with a sialidase-deficient mutant reduced free sialic acid levels and thereby impaired the expansion of *C. difficile*.

The decreased severity of DSS-induced colitis in mice treated with sialidase inhibitor demonstrated the contribution of sialic acid in *E. coli* expansion and in the ensuing inflammatory response. Administration of free sialic acid to mice before or during DSS challenge, however, failed to affect the levels of intestinal *E. coli* and the severity of colitis (Supplementary Fig. 5). Considering that monosaccharides are absorbed in the small intestine and only minute amounts reach the colon, oral supplementation with sialic acid is thus unlikely to influence the outgrowth of *E. coli* in the colon. Therefore, the release of sialic acid from host glycans is critical in promoting the growth advantage of *E. coli*. The increase in sialylation of intestinal mucins during colitis^35^,^36^ likely facilitates the local release of free sialic acid during inflammation.

Our findings showed that commensal *E. coli* was a potent activator of a pro-inflammatory response in intestinal DCs. The activation of DCs was likely induced by surface LPSs triggering Toll-like receptor-4 signalling. The strong pyrogenic effect of commensal *E. coli* over the one induced by *B. thetaiotaomicron* matches previous findings showing that *E. coli* LPS was more active than *Bacteroides* spp.-derived LPS at inducing TNF-α production^37^. Similar differences in pyrogenicity were also noted in mouse models^38^,^39^. These results demonstrate that increased *E. coli* levels likely exacerbate inflammation through activation of mucosal immune cells such as DCs. Several intestinal bacteria regulate mucosal immunity, thereby affecting the occurrence of Th17 cells in the lamina propria in the case of segmented filamentous bacteria^40^,^41^, or the induction of Foxp3^+^ Treg cells in the case of a group of *Clostridium* spp.^42^ and *B. fragilis*^43^. We cannot exclude that the disappearance of inflammation-lessening bacteria also affects the severity of colitis alongside the expansion of pro-inflammatory *E. coli*, but such contributions are unlikely since we failed to detect any differences in the distribution and amounts of mucosal immune cells between WT and ST mice before DSS-induced colitis^18^. Therefore, we conclude that the proliferation of *E. coli* supported by α2,3-linked sialic acids was the main factor regulating the magnitude of intestinal inflammation triggered by DSS ingestion.

This study demonstrated the critical role of α2,3-linked sialic acids provided by milk-derived 3SL during lactation and by host mucosal glycans in establishing an intestinal niche for *E. coli* in mice. Expansion of *E. coli* during colitis directly depended on sialic acid release from host glycans after sialidase activity. This resulting overgrowth of *E. coli* leads to exacerbation of the pro-inflammatory response by intestinal DCs. The beneficial outcome of sialidase inhibition on the severity of DSS-induced colitis suggests that sialidase inhibitors should be investigated as agents able to reduce intestinal inflammation by preventing dysbiosis manifested by *Enterobacteriaceae* expansion.

## Methods

### Bacterial DNA extraction and quantitative PCR

DNA was isolated from faecal samples using the QIAamp DNA stool mini kit (Qiagen) according to manufacturer's instructions. Lysis temperature was increased to 95 °C for 5 min to ensure complete cell lysis of Gram-positive cells. The proportion of bacterial family and genera in faecal samples were determined by real-time PCR using the EvaGreen qPCR Master Mix (Biotium). Cycling conditions were 40 cycles at 95 °C for 10 s, 60 °C for 10 s and 72 °C for 25 s after an initial denaturation at 95 °C for 3 min. Primer pairs specific for 16S rRNA of *Bacteroidetes* (Bac32F: 5′-AACGCTAGCTACAGGCTT-3′, Bac303R: 5′-CCAATGTGGGGGACCTTC-3′); *Enterobacteriaceae* (Eco1457F: 5′-CATTGACGTTACCCGCAGAAGAAGC-3′, Eco1652R: 5′-CTCTACGAGACTCAAGCTTGC-3′); and total bacteria (Eub338F: 5′-ACTCCTACGGGAGGCAGCAG-3′, Eub518R: 5′-ATTACCGCGGCTGCTGG-3′) were described previously^44^,^45^. Primers (UAL1939b: 5′-ATGGAATTTCGCCGATTTTGC-3′, UAL2105b: 5′-ATTGTTTGCCTCCCTGCTGC-3′) targeting β-glucuronidase *uidA* gene was used to evaluate the relative abundance of *E. coli*^22^. Quantification values were calculated by the 2^−▵Ct^ method relative to total bacterial 16S rRNA amplicons^46^.

### 16S rRNA pyrosequencing

Faecal DNA was isolated from fresh stool samples of 7-week-old WT and ST male mice before and at day 8 after initiation of DSS treatment. The 16S rRNA V5–V6 region was amplified from faecal DNA samples using primer 784F and 1061R^47^. Amplicons were sequenced using a Roche 454 GS-FLX system (DNAVision, Belgium). The QIIME software was used for taxonomic classification. Taxonomy was assigned using Ribosomal Database Project (RDP) classifier and the Greengenes database^48^. Bacterial diversity was determined at the phylum, family and genus levels. The sequencing reads have been deposited in the NCBI sequence read archive SRA as Bioproject PRJNA289738.

### Mouse models

WT and sialyltransferase *St3gal4*^−/−^ mice^49^ were of C57BL/6 background and derived from the same breed and maintained in light-cycled and climate-controlled facility. Animals were received regular laboratory chow diet (KLIBA extrudat no. 3436, Provimi Kliba, SA, Switzerland) and sterile water *ad libitum*. Synchronized matings were set up for WT and ST mice to allow the exchange of newborn mice for cross-fostering experiments. All experiments were performed in compliance with the Swiss Animal Protection Ordinance and approved by the Veterinary Office of the Canton of Zurich, Switzerland.

### Antibiotic treatment and DSS-induced colitis

Six- to 7-week-old male mice were treated with 3–3.5% (w/v) DSS (molecular weight=36–50 kDa; MP Biomedicals) in drinking water for 5 days, followed by a supply of normal water until sacrifice of the animals. Body weight and physical activity were monitored daily. For long-term antibiotic treatment, 3-week-old mice were provided with sterile drinking water supplemented with vancomycin (0.5 g l^−1^), streptomycin (1 g l^−1^), neomycin (1 g l^−1^), chloramphenicol (0.5 g l^−1^) or metronidazole (1 g l^−1^) plus aspartame (0.25%, w/v) for 3 weeks before the beginning of DSS treatment. For short-term antibiotic treatment, mice were administered sterile drinking water supplemented with vancomycin (0.5 g l^−1^) or streptomycin (1 g l^−1^) for 8 days, during which they also received 3% of DSS on the first 5 days.

### Transepithelial permeability assay

Mice were gavaged with 600 mg kg^−1^ body weight of FITC–dextran (MW 3000–5000, Sigma-Aldrich) and whole blood was collected by cardiac puncture 4 h after gavage. Blood serum was collected after centrifugation at 1500*g* for 10 min. Serum fluorescence intensity was measured using a multi-detection microplate reader (Tecan Infinite M200 Pro, Switzerland) with an excitation wavelength of 485 nm and an emission wavelength of 535 nm. FITC concentration (μg ml^−1^) was calculated from a standard curve using serial dilutions of FITC–dextran^50^.

### Bacterial strains and mutant construct

*E. coli* strain EHV2 was isolated from inflamed colon surface of DSS-treated C57/BL6 mice. The isolate strain was confirmed by 16S rRNA sequencing, universal stress protein *uspA*^23^, gyrase *gyrB*^24^ sequencing and phenotypic analysis (API-20E *Enterobacteriaceae* identification kit; bioMerieux). *E. coli* strain HS996 was obtained from Genebridge and transformed with pET16b vector containing an ampicillin resistance gene. HS996-*nanT* knockout mutant was constructed by introducing a kanamycin-resistance cassette by homologous recombination into the nanT locus with followed by manufacture's instruction (Genebridge, Germany). The genomic primers used for the targeting construct were designed according to the flanking regions of the *E. coli nanT* gene: forward primer f182 was 5′-ATACCAAAGCGTGTGGGCATCGCCCACCGCGGGAGACTCACAATGAGTACAATTAACCCTCACTAAAGGGCG-3′, and reverse primer r1723 was 5′-GCAACAGGATTAACTTTTGGTTTTGACTAAATCGTTTTTGGCGCTGCCAATAATACGACTCACTATAGGGCTC-3′. Null mutation at the *nanT* locus was confirmed by genome sequencing and growth phenotype. *B. thetaiotaomicron* (DSM 2079^T^) was obtained from the German Collection of Microorganisms and Cell Cultures (Braunschweig, Germany). Cells were grown in YCFA (yeast extract-casein hydrolysate-fatty acids) medium containing volatile fatty acids^51^ at 37 °C anaerobically in rubber-sealed Hungate tubes. Cell density was measured by a spectrophotometer at 600 nm (S2100 Diode array, Biochrom WPA).

### Carbohydrate metabolism assay

*E. coli* EHV2 (10^7^ cells) were cultured in 3 ml of M9 minimal medium^52^ containing 10 mM of either glucose (Glc), galactose (Gal), *N*-acetylglucosamine (GlcNAc), *N*-acetylgalactosamine (GalNAc), fucose (Fuc) and *N*-acetylneuraminic acid (Sia) as single carbohydrate source at 37 °C for 24 h. The milk oligosaccharides 3SL and 6SL were tested as 5 mM in 3 ml of M9 minimal medium supplemented with PBS or sterile-filtered mouse caecal fluid (1.5%, v/v). All neutral monosaccharides were purchased from Sigma-Aldrich except *N*-acetylneuraminic acid from Carbosynth (Berkshire, UK). The oligosaccharides 3SL and 6SL were obtained from Glycom A/S (Lyngby, Denmark). Cell density was determined by OD 600 values at 3, 6, 12 and 24 h. For determination of the growth phenotype in nanT mutant and ManX mutant, parental and mutant strains were cultured in modified M9 minimal medium (additional 0.003% L-histidine, 0.004% leucine and 0.01% yeast extract) containing 10 mM of glucose or *N*-acetylneuraminic acid.

### Sialidase activity assay

Mouse caecal content was collected and centrifuged at 15,000*g* for 10 min at 4 °C. The supernatant was filtered through a 0.45-μm membrane to yield caecal fluid. The fluorogenic substrate 2′-(4-methylumbelliferyl)-α-D-*N*-acetylneuraminic acid sodium salt (4-MU-NeuNAc; Carbosynth) was used to determine sialidase activity. In brief, caecal fluid (10%, v/v) was incubated with 0.1 mM 4-MU-NeuNAc in 0.2 ml of 100 mM sodium acetate buffer (pH 7.4) at 37 °C for 15 min. Assays were stopped by adding 0.8 ml of 0.5 M sodium carbonate buffer (pH 10.5) and further diluted 20-fold before fluorescence measurement. Cleaved 4-methylumbelliferone (4-MU) was measured by fluorescence detection in a multi-detection microplate reader at an excitation wavelength of 360 nm and an emission wavelength of 440 nm (ref. 53).

### Quantitative PCR of bacterial sialidase genes

Sequences of sialidase (EC 3.2.1.18) genes from *Bacteroidaceae* were retrieved from the GH33 sialidase family of the CAZy database. Primers encompassing conserved DNA stretches of sialidase genes from *P. distasonis*, *B. vulgatus*, *B. thetaiotaomicron* and *B. fragilis* were designed based on multiple alignment analysis. The lack of significant sequence similarity of the selected primers with unrelated bacterial sequences was confirmed by BLAST analysis. The *B. vulgatus* sialidase primers used for quantitative PCR analysis were Bv-f266: 5′-GGAGGGGAAAGACTTATTTTGC-3′, Bv-r501: 5′-TTCCACCACTTCTGCCGAC-3′; cycling conditions were 40 cycles at 95 °C for 10 s, 60 °C for 10 s and 72 °C for 25 s after an initial denaturation at 95 °C for 3 min. Quantification values are represented as gene copy numbers per μg of total faecal DNA.

### Molecular cloning and purification of sialidase

The gene encoding *B. vulgatus*_4143 sialidase (Gene ID: 5305102) was amplified by PCR using the genomic DNA from caecum sample in WT–DSS mouse as template. *Nde*I and *Bam*HI sites were introduced in the forward Bvu_4143F 5′-GGCCATATGAGAAACCCTAGCTTATTA-3′ and reverse primer Bvu_4143R: 5′- GCGGGATCCTTATTTGGTCTTAATAAT-3′, respectively. PCR conditions were thirty cycles of 30 s at 95 °C, 30 s at 53 °C, 3.5 min at 72 °C. The PCR product was digested with *Nde*I and *Bam*HI and subcloned into the pET16b expression vector (Novagen). The pET16b- BVU_4143 vector was transformed into *E. coli* BL21-star (DE3) cells (Invitrogen) cultured in LB broth and supplemented with ampicillin (100 μg ml^−1^) at 37 °C. On reaching an OD 600 value of 0.5, BVU_4143 expression was induced by adding 0.5 mM Isopropyl β-D-1-thiogalactopyranoside and incubating bacteria at 30 °C for 5 h. Bacteria were then resuspended in 100 mM Tris-HCl plus 20 mM imidazole (pH 7.4) and disrupted by sonication. The resulting cell extract was incubated with Ni-sepharose (GE Healthcare Life Sciences) at 4 °C overnight, washed with 100 mM Tris-HCl containing 40 mM imidazole and the His_6_-tagged BVU_4143 sialidase was eluted with 500 mM imidazole.

### Bacterial colonization fitness assay

The relative fitness of *E. coli* strains for colonization of the mouse intestine was monitored as described^34^. Briefly, 6-week-old WT mice were given drinking water containing ampicillin (2 mg ml^−1^) for 2 days before gavage with 10^6^ c.f.u. of parental HS996-amp^r^ and HS996-nanT mutant::Kana^r^ *E. coli*. Fresh faecal samples were collected, serially diluted and plated on LB agar containing ampicillin (100 μg ml^−1^) or ampicillin plus kanamycin (30 μg ml^−1^) by days 1, 2, 5 and 10 after gavage. Competitive index was calculated as the ratio of parental to *nanT* mutant *E. coli*.

### Quantification of caecal sialic acids

Mouse caecal content (∼500 mg) was weighed out and centrifuged for 10 min at 16,000*g* at 4 °C. The supernant was collected and filtered through 0.45 μm cronus HPLC membrane to get caecal fluid and stored at −20 °C before use. Caecal fluid was derivatized with 1,2-diamino-4,5-methylene-dioxybezene (DMB; Sigma-Aldrich) as described previously^54^. In brief, 10 μl of caecal fluid was incubated with 200 μl of the DMB solution at 50 °C for 2.5 h in the dark. DMB solution was prepared by dissolving DMB dihydrochloride (7 mM) in 1.4 M acetic acid containing 0.75 M β-mercaptoethanol and 18 mM sodium hydrosulfite. The reaction was stopped by adding 800 μl of ice-cold distilled water. The derivatized product was analysed by reverse-phase HPLC using a ODS Hypersil 150 × 3 mm column (Thermo scientific). The mobile phase was acetonitrile/methanol/water (9:7:84, v/v) at a flow rate of 0.3 ml min^−1^. Florescence of the derivatized product was monitored at 373 nm (excitation) and 448 nm (emission). DMB-derivatized Neu5Ac was identified by comparison with authentic sialic acid standards.

### Sialidase inhibition

The sialidase inhibitor *N*-acetyl-2,3-didehydro-2-deoxyneuraminic acid (Neu5Ac2en) was prepared in house based on published procedures^55^. For *in vitro* inhibition, *E. coli* EHV2 was cultured for 24 h at 37 °C in M9 minimal media containing 5 mM 3SL, caecal fluid (1.5%, v/v) and varying Neu5Ac2en concentrations. For *in vivo* inhibition, mice were anesthetized by isoflurane inhalation, gavaged with 300 μl of Neu5Ac2en (10 mg kg^−1^ per day) in sterile saline at days 0, 1, 2 and 5 of DSS challenge. Control groups received sterile saline.

### Histological staining of colonic tissue

Distal colons were removed, cut longitudinally, and fixed in 10% neutral buffered formalin then embedded in paraffin. Tissue samples were cut in serial 5-μm sections, which were stained with hematoxylin-eosin (Sigma-Aldrich). Histological sections were examined by using microscope Zeiss Axio Imager.Z2, objective Zeiss EC Plan Neofluar 10 × /0.3. Image was acquired by Zeiss AxioCam HrC camera and analysed with Zeiss AxioVision software (AxioVs40V4.8.2.0). Sections were scored individually by an independent investigator blinded to the type of treatment. Morphological changes and leukocyte infiltration in the colon were scored as previously described^56^. Histology was scored as follows: epithelium 0: normal morphology; 1: loss of goblet cells; 2: loss of goblet cells in large areas; 3: loss of crypts; and 4: loss of crypts in large areas. Infiltration 0: no infiltrate; 1: infiltrate around crypt basis; 2: infiltrate reaching to *lamina muscularis mucosae*; 3: extensive infiltration reaching the *lamina muscularis mucosae* and thickening of the mucosa with abundant oedema; and 4: infiltration of the *lamina submucosa*. The total score represents the sum of the epithelium and infiltration score.

### DC Isolation and stimulation

Mesenteric lymph nodes were isolated and incubated in 2.5 mg ml^−1^of collagenase type D (Roche) in RPMI 1640 containing 10% FCS for 10 min at 37 °C. Tissues were gently homogenized by passing through an 18-gauge needle, then incubated for 30 min at 37 °C. The resulting cell suspensions were filtered through 40-μm cell strainers and incubated with Fc-blocker (anti-CD16/32; eBioscience) for 10 min. CD11c^+^ cells were isolated with anti-CD11c MicroBeads (Miltenyi Biotec) according to the manufacturer's instructions. CD11c^+^ DCs (2 × 10^5^ cells per ml) were culture in RPMI 1640 containing 10% FCS, and stimulated with fixed bacteria or PBS for 14 h at 37 °C. *E. coli* (EHV2) and *B. thetaiotaomicron* (DSMZ 2079T) were fixed in 0.5% paraformaldehyde for 15 min at room temperature and washed with PBS before stimulation. Fixed bacteria were co-cultured with DCs in a ratio 100:1. After stimulation, DCs were stained with fluorochrome-labeled anti-mouse antibodies: MHC-II–FITC (BD Biosciences), CD86-PE (eBioscience), CD40-PE-Cy7 (BioLegend) and CD11C-APC (BD Biosciences) for 30 min at 4 °C. Cells were analysed by flow cytometry (FACSCanto II). For monocytes stimulation, human THP-1 cells (1 × 10^6^ cells per ml) were stimulated with bacteria or PBS for 14 h as described above, but in bacteria to cells ratios of 5:1 and 1:1. After stimulation, THP-1 cells were stained with CD54-PE (BD Biosciences) for 30 min at 4 °C and analysed by flow cytometry. The supernatants of stimulated DCs were collected and analysed for IL-6, IL-10, IL-12p40 and TNF-α cytokine production by multiplex bead array (Cytolab AG, Switzerland).

### Statistical analysis

Results are presented as mean±s.e.m. unless specified. Difference between groups was analysed by unpaired Student's *t*-test (two-tailed) and one-way analysis of variance (ANOVA) with Bonferroni's multiple comparison post-test using GraphPad Prism 5. *P* values below 0.05 were considered significant.

## Additional information

**How to cite this article:** Huang, Y. L. *et al.* Sialic acid catabolism drives intestinal inflammation and microbial dysbiosis in mice. *Nat. Commun.* 6:8141 doi: 10.1038/ncomms9141 (2015).

## Supplementary Material

Supplementary Information Supplementary Figures 1-5, Supplementary Methods and Supplementary Reference

## References

[b1] Wang Y. *et al.* 16S rRNA gene-based analysis of fecal microbiota from preterm infants with and without necrotizing enterocolitis. ISME J. 3, 944–954 (2009). 19369970 10.1038/ismej.2009.37 PMC2713796

[b2] Kamada N., Seo S. U., Chen G. Y. & Nunez G. Role of the gut microbiota in immunity and inflammatory disease. Nat. Rev. Immunol. 13, 321–335 (2013). 23618829 10.1038/nri3430

[b3] Zhao L. The gut microbiota and obesity: from correlation to causality. Nat. Rev. Microbiol. 11, 639–647 (2013). 23912213 10.1038/nrmicro3089

[b4] Oertli M. *et al.* Helicobacter pylori gamma-glutamyl transpeptidase and vacuolating cytotoxin promote gastric persistence and immune tolerance. Proc. Natl Acad. Sci. USA 110, 3047–3052 (2013). 23382221 10.1073/pnas.1211248110 PMC3581963

[b5] Scher J. U. & Abramson S. B. The microbiome and rheumatoid arthritis. Nat. Rev. Rheumatol. 7, 569–578 (2011). 21862983 10.1038/nrrheum.2011.121 PMC3275101

[b6] Louis P., Hold G. L. & Flint H. J. The gut microbiota, bacterial metabolites and colorectal cancer. Nat. Rev. Microbiol. 12, 661–672 (2014). 25198138 10.1038/nrmicro3344

[b7] El Kaoutari A., Armougom F., Gordon J. I., Raoult D. & Henrissat B. The abundance and variety of carbohydrate-active enzymes in the human gut microbiota. Nat. Rev. Microbiol. 11, 497–504 (2013). 23748339 10.1038/nrmicro3050

[b8] Chassard C. & Lacroix C. Carbohydrates and the human gut microbiota. Curr. Opin. Clin. Nutr. Metab. Care. 16, 453–460 (2013). 23719143 10.1097/MCO.0b013e3283619e63

[b9] Zivkovic A. M., German J. B., Lebrilla C. B. & Mills D. A. Human milk glycobiome and its impact on the infant gastrointestinal microbiota. Proc. Natl Acad. Sci. USA 108, 4653–4658 (2011). 20679197 10.1073/pnas.1000083107 PMC3063602

[b10] Marcobal A. *et al.* Consumption of human milk oligosaccharides by gut-related microbes. J. Agric. Food. Chem. 58, 5334–5340 (2010). 20394371 10.1021/jf9044205 PMC2866150

[b11] Pacheco A. R. *et al.* Fucose sensing regulates bacterial intestinal colonization. Nature 492, 113–117 (2012). 23160491 10.1038/nature11623 PMC3518558

[b12] Bouchet V. *et al.* Host-derived sialic acid is incorporated into Haemophilus influenzae lipopolysaccharide and is a major virulence factor in experimental otitis media. Proc. Natl Acad. Sci. USA 100, 8898–8903 (2003). 12855765 10.1073/pnas.1432026100 PMC166410

[b13] Harvey H. A., Swords W. E. & Apicella M. A. The mimicry of human glycolipids and glycosphingolipids by the lipooligosaccharides of pathogenic neisseria and haemophilus. J. Autoimmun. 16, 257–262 (2001). 11334490 10.1006/jaut.2000.0477

[b14] Carlin A. F., Lewis A. L., Varki A. & Nizet V. Group B streptococcal capsular sialic acids interact with siglecs (immunoglobulin-like lectins) on human leukocytes. J. Bacteriol. 189, 1231–1237 (2007). 16997964 10.1128/JB.01155-06 PMC1797352

[b15] Aspholm M. *et al.* SabA is the *H. pylori* hemagglutinin and is polymorphic in binding to sialylated glycans. PLoS Pathog. 2, e110 doi:10.1371/journal.ppat.0020110 (2006). 17121461 10.1371/journal.ppat.0020110 PMC1626103

[b16] Byres E. *et al.* Incorporation of a non-human glycan mediates human susceptibility to a bacterial toxin. Nature 456, 648–652 (2008). 18971931 10.1038/nature07428 PMC2723748

[b17] Kashyap P. C. *et al.* Genetically dictated change in host mucus carbohydrate landscape exerts a diet-dependent effect on the gut microbiota. Proc. Natl Acad. Sci. USA 110, 17059–17064 (2013). 24062455 10.1073/pnas.1306070110 PMC3800993

[b18] Fuhrer A. *et al.* Milk sialyllactose influences colitis in mice through selective intestinal bacterial colonization. J. Exp. Med. 207, 2843–2854 (2010). 21098096 10.1084/jem.20101098 PMC3005226

[b19] Kurakevich E., Hennet T., Hausmann M., Rogler G. & Borsig L. Milk oligosaccharide sialyl(alpha2,3)lactose activates intestinal CD11c+ cells through TLR4. Proc. Natl Acad Sci USA 110, 17444–17449 (2013). 24101501 10.1073/pnas.1306322110 PMC3808656

[b20] Lupp C. *et al.* Host-mediated inflammation disrupts the intestinal microbiota and promotes the overgrowth of Enterobacteriaceae. Cell Host Microbe 2, 204 (2007). 18030708 10.1016/j.chom.2007.08.002

[b21] Schuppler M., Lotzsch K., Waidmann M. & Autenrieth I. B. An abundance of Escherichia coli is harbored by the mucosa-associated bacterial flora of interleukin-2-deficient mice. Infect. Immun. 72, 1983–1990 (2004). 15039318 10.1128/IAI.72.4.1983-1990.2004 PMC375167

[b22] Maheux A. F. *et al.* Analytical comparison of nine PCR primer sets designed to detect the presence of Escherichia coli/Shigella in water samples. Water Res. 43, 3019–3028 (2009). 19482328 10.1016/j.watres.2009.04.017

[b23] Chen J. & Griffiths M. W. PCR differentiation of Escherichia coli from other gram-negative bacteria using primers derived from the nucleotide sequences flanking the gene encoding the universal stress protein. Lett. Appl. Microbiol. 27, 369–371 (1998). 9871356 10.1046/j.1472-765x.1998.00445.x

[b24] Fukushima M., Kakinuma K. & Kawaguchi R. Phylogenetic analysis of Salmonella, Shigella, and Escherichia coli strains on the basis of the gyrB gene sequence. J. Clin. Microbiol. 40, 2779–2785 (2002). 12149329 10.1128/JCM.40.8.2779-2785.2002 PMC120687

[b25] Kim S., Oh D. B., Kang H. A. & Kwon O. Features and applications of bacterial sialidases. Appl. Microbiol. Biotechnol. 91, 1–15 (2011). 21544654 10.1007/s00253-011-3307-2

[b26] Varki A. & Gagneux P. Multifarious roles of sialic acids in immunity. Ann. NY Acad. Sci. 1253, 16–36 (2012). 22524423 10.1111/j.1749-6632.2012.06517.x PMC3357316

[b27] Roy S., Douglas C. W. & Stafford G. P. A novel sialic acid utilization and uptake system in the periodontal pathogen Tannerella forsythia. J. Bacteriol. 192, 2285–2293 (2010). 20190043 10.1128/JB.00079-10 PMC2863479

[b28] Nagalingam N. A. & Lynch S. V. Role of the microbiota in inflammatory bowel diseases. Inflamm. Bowel. Dis. 18, 968–984 (2012). 21936031 10.1002/ibd.21866

[b29] Winter S. E. *et al.* Host-derived nitrate boosts growth of E. coli in the inflamed gut. Science 339, 708–711 (2013). 23393266 10.1126/science.1232467 PMC4004111

[b30] Singh V. *et al.* Interplay between enterobactin, myeloperoxidase and lipocalin 2 regulates E. coli survival in the inflamed gut. Nat. Commun. 6, 7113 (2015). 25964185 10.1038/ncomms8113 PMC6336494

[b31] Chang D. E. *et al.* Carbon nutrition of Escherichia coli in the mouse intestine. Proc. Natl Acad. Sci. USA 101, 7427–7432 (2004). 15123798 10.1073/pnas.0307888101 PMC409935

[b32] Fabich A. J. *et al.* Comparison of carbon nutrition for pathogenic and commensal Escherichia coli strains in the mouse intestine. Infect. Immun. 76, 1143–1152 (2008). 18180286 10.1128/IAI.01386-07 PMC2258830

[b33] Gophna U., Sommerfeld K., Gophna S., Doolittle W. F. & Veldhuyzen van Zanten S. J. Differences between tissue-associated intestinal microfloras of patients with Crohn's disease and ulcerative colitis. J. Clin. Microbiol. 44, 4136–4141 (2006). 16988016 10.1128/JCM.01004-06 PMC1698347

[b34] Ng K. M. *et al.* Microbiota-liberated host sugars facilitate post-antibiotic expansion of enteric pathogens. Nature 502, 96–99 (2013). 23995682 10.1038/nature12503 PMC3825626

[b35] Parker N., Tsai H. H., Ryder S. D., Raouf A. H. & Rhodes J. M. Increased rate of sialylation of colonic mucin by cultured ulcerative colitis mucosal explants. Digestion 56, 52–56 (1995). 7895933 10.1159/000201222

[b36] Campbell B. J., Yu L. G. & Rhodes J. M. Altered glycosylation in inflammatory bowel disease: a possible role in cancer development. Glycoconj. J. 18, 851–858 (2001). 12820718 10.1023/a:1022240107040

[b37] Delahooke D. M., Barclay G. R. & Poxton I. R. A re-appraisal of the biological activity of bacteroides LPS. J. Med. Microbiol. 42, 102–112 (1995). 7869345 10.1099/00222615-42-2-102

[b38] Takada H. *et al.* Bacteroides lipopolysaccharides (LPS) induce anaphylactoid and lethal reactions in LPS-responsive and -nonresponsive mice primed with muramyl dipeptide. J. Infect. Dis. 162, 428–434 (1990). 2197336 10.1093/infdis/162.2.428

[b39] Poxton I. R. & Edmond D. M. Biological activity of Bacteroides lipopolysaccharide—reappraisal. Clin. Infect. Dis. 20, S149–S153 (1995). 7548538 10.1093/clinids/20.supplement_2.s149

[b40] Ivanov I. I. *et al.* Induction of intestinal Th17 cells by segmented filamentous bacteria. Cell 139, 485–498 (2009). 19836068 10.1016/j.cell.2009.09.033 PMC2796826

[b41] Gaboriau-Routhiau V. *et al.* The key role of segmented filamentous bacteria in the coordinated maturation of gut helper T cell responses. Immunity 31, 677–689 (2009). 19833089 10.1016/j.immuni.2009.08.020

[b42] Atarashi K. *et al.* Induction of colonic regulatory T cells by indigenous Clostridium species. Science 331, 337–341 (2011). 21205640 10.1126/science.1198469 PMC3969237

[b43] Mazmanian S. K., Round J. L. & Kasper D. L. A microbial symbiosis factor prevents intestinal inflammatory disease. Nature 453, 620–625 (2008). 18509436 10.1038/nature07008

[b44] Bernhard A. E. & Field K. G. Identification of nonpoint sources of fecal pollution in coastal waters by using host-specific 16S ribosomal DNA genetic markers from fecal anaerobes. Appl. Environ. Microbiol. 66, 1587–1594 (2000). 10742246 10.1128/aem.66.4.1587-1594.2000 PMC92027

[b45] Fierer N., Jackson J. A., Vilgalys R. & Jackson R. B. Assessment of soil microbial community structure by use of taxon-specific quantitative PCR assays. Appl. Environ. Microbiol. 71, 4117–4120 (2005). 16000830 10.1128/AEM.71.7.4117-4120.2005 PMC1169028

[b46] Schmittgen T. D. & Livak K. J. Analyzing real-time PCR data by the comparative C(T) method. Nat. Protoc. 3, 1101–1108 (2008). 18546601 10.1038/nprot.2008.73

[b47] Andersson A. F. *et al.* Comparative analysis of human gut microbiota by barcoded pyrosequencing. PLoS ONE 3, e2836 (2008). 18665274 10.1371/journal.pone.0002836 PMC2475661

[b48] De Filippo C. *et al.* Impact of diet in shaping gut microbiota revealed by a comparative study in children from Europe and rural Africa. Proc. Natl Acad. Sci. USA 107, 14691–14696 (2010). 20679230 10.1073/pnas.1005963107 PMC2930426

[b49] Ellies L. G. *et al.* Sialyltransferase ST3Gal-IV operates as a dominant modifier of hemostasis by concealing asialoglycoprotein receptor ligands. Proc. Natl Acad. Sci. USA 99, 10042–10047 (2002). 12097641 10.1073/pnas.142005099 PMC126621

[b50] Napolitano L. M., Koruda M. J., Meyer A. A. & Baker C. C. The impact of femur fracture with associated soft tissue injury on immune function and intestinal permeability. Shock 5, 202–207 (1996). 8696984 10.1097/00024382-199603000-00006

[b51] Duncan S. H., Hold G. L., Harmsen H. J., Stewart C. S. & Flint H. J. Growth requirements and fermentation products of Fusobacterium prausnitzii, and a proposal to reclassify it as Faecalibacterium prausnitzii gen. nov., comb. nov. Int. J. Syst. Evol. Microbiol. 52, 2141–2146 (2002). 12508881 10.1099/00207713-52-6-2141

[b52] Sambrook J. & Russell D. Molecular Cloning: A Laboratory Manual Cold Spring Harbor Laboratory Press (2001).

[b53] Thompson H., Homer K. A., Rao S., Booth V. & Hosie A. H. F. An orthologue of Bacteroides fragilis NanH is the principal sialidase in Tannerella forsythia. J. Bacteriol. 191, 3623–3628 (2009). 19304852 10.1128/JB.01618-08 PMC2681896

[b54] Hara S. *et al.* Determination of mono-O-acetylated N-acetylneuraminic acids in human and rat sera by fluorometric high-performance liquid chromatography. Anal. Biochem. 179, 162–166 (1989). 2757191 10.1016/0003-2697(89)90218-2

[b55] von Itzstein M., Wu W. Y. & Jin B. The synthesis of 2,3-didehydro-2,4-dideoxy-4-guanidinyl-N-acetylneuraminic acid: a potent influenza virus sialidase inhibitor. Carbohydr. Res. 259, 301–305 (1994). 8050102 10.1016/0008-6215(94)84065-2

[b56] Hausmann M. *et al.* *In vivo* treatment with the herbal phenylethanoid acteoside ameliorates intestinal inflammation in dextran sulphate sodium-induced colitis. Clin. Exp. Immunol. 148, 373–381 (2007). 17437425 10.1111/j.1365-2249.2007.03350.x PMC1868873

