# Supplementary material for: Sialic acid catabolism drives intestinal inflammation and microbial dysbiosis in mice
Source: Nat Commun. 2015 Aug 25;6:8141. doi: 10.1038/ncomms9141 (PMC4560832; doi:10.1038/ncomms9141)
Supplement: Supplementary Information — Supplementary Figures 1-5, Supplementary Methods and Supplementary Reference [file ncomms9141-s1.pdf]

Supplementary Information

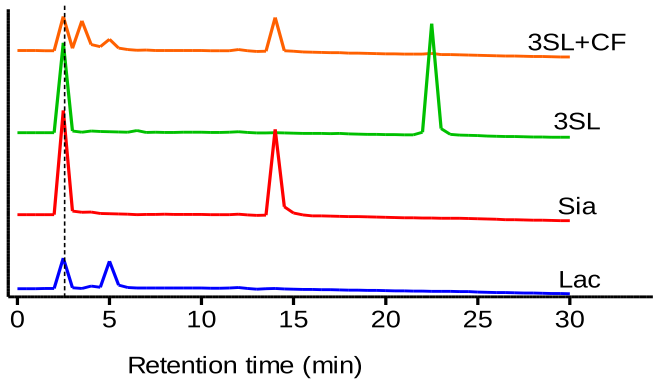

Supplementary Figure 1. Specificity of mouse caecum fluid sialidase. Cleavage of sialic acid from 3SL after incubation with caecum fluid for 16 h at 37°C. Reaction products were separated by HPLC using a CarboPac PA200 column (Dionex). Carbohydrates were identified by comparison with authentic standards. Lac, lactose; Sia, *N*-acetylneuraminic acid. Representative chromatograms from two independent experiments are shown.

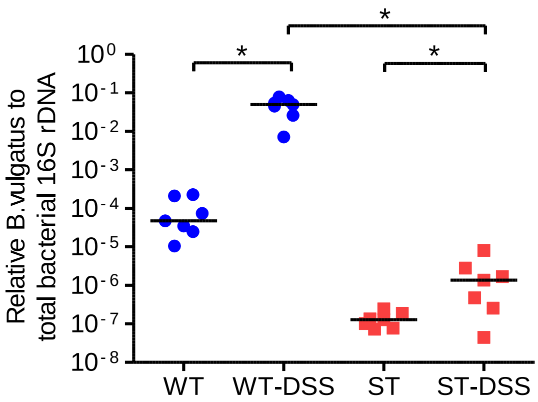

Supplementary Figure 2. Relative abundance of *B. vulgatus* in the colon of mice. The relative abundance of *B. vulgatus* was determined by real-time PCR using TaqMan assay. Each data point represents a single mouse from two independent experiments, N=6-7, *$p < 0.05$ (Two-tailed Student's t-test).

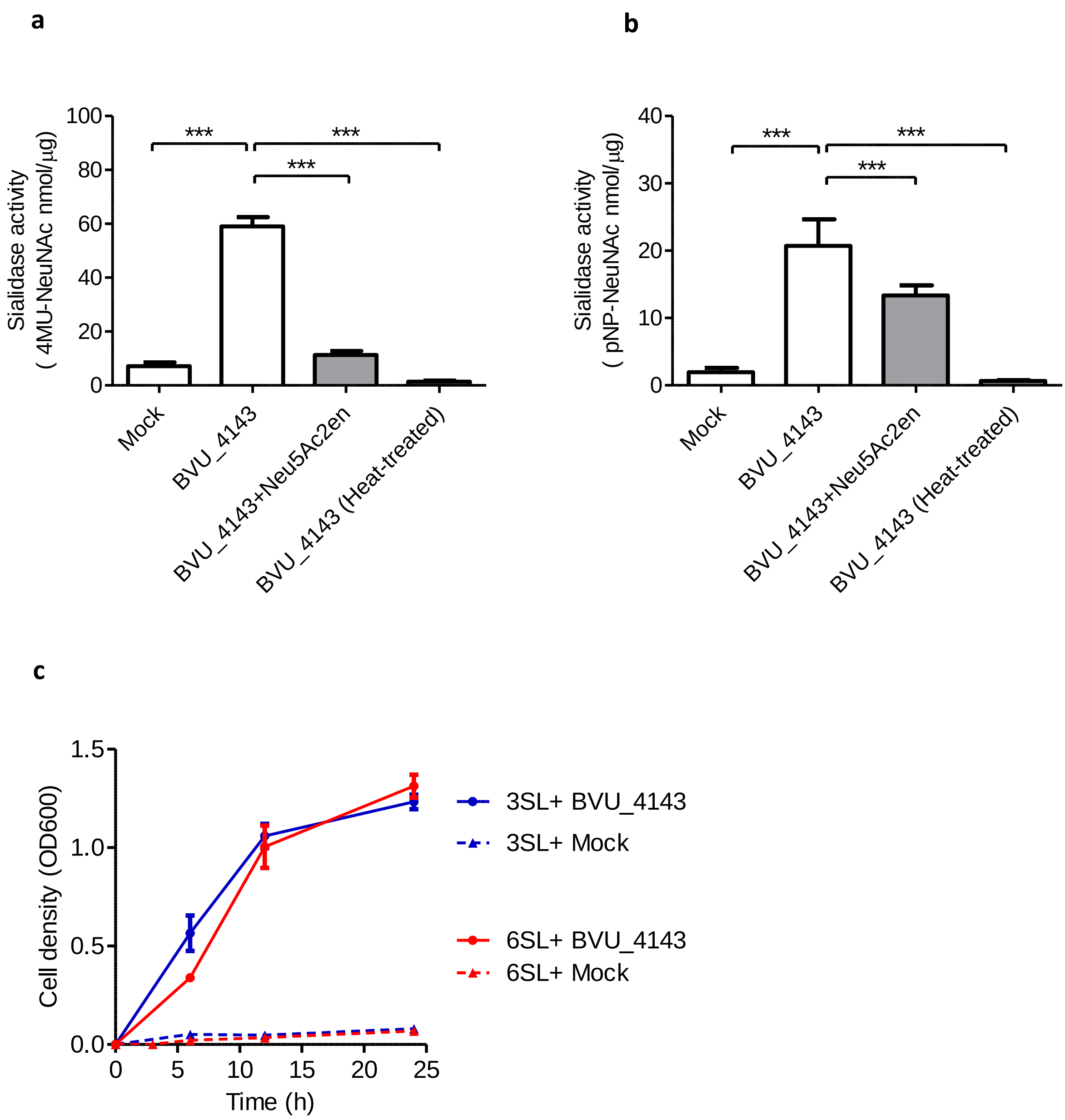

Supplementary Figure 3. Characterization of *B. vulgatus* BVU_4143 sialidase. (a) Recombinant BVU_4143 was purified as His-tagged protein. Sialidase activity of recombinant protein was determined by the hydrolysis of 4-MU-NeuNAc and **(b)** pNP-NeuNAc. For sialidase inhibition, B. vul sialidase was pretreated with Neu5Ac2en for 20 min with final concentration 200 µM. Heat, heat inactivation at 95 °C for 10 min. **(c)** Growth of *E. coli* EHV2 in minimal medium containing 5 mM of 3SL and 6SL with and without supplementation of recombinant sialidase. Data represent as mean ± SEM, N=4, ***p < 0.001 (Two-tailed Student's t-test).

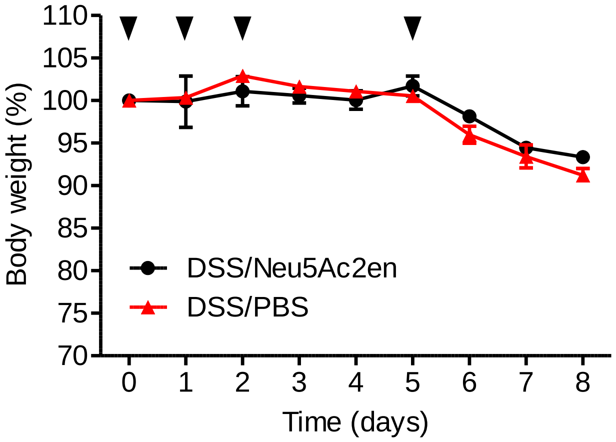

Supplementary Figure 4. Administration of sialidase inhibitor in ST mice. ST mice were administrated with PBS or Neu5Ac2en (10 mg/kg/day) at indicated time points (arrowhead) during DSS (3%) challenge for 5 days. Relative change in body weight in ST mice challenged with DSS was monitored daily, N=4.

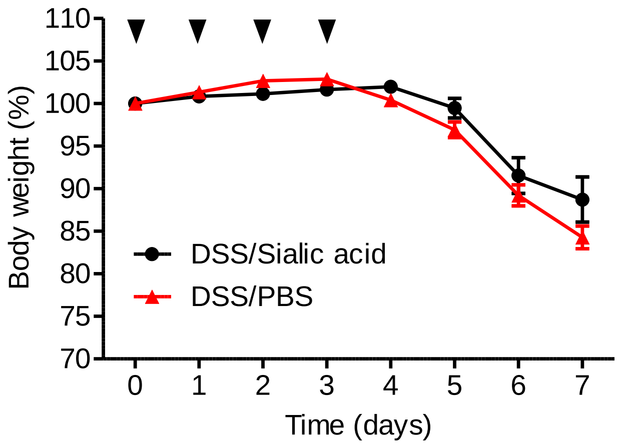

Supplementary Figure 5. Oral administration of sialic acid in WT mice. WT mice were orally gavaged with 300 µl of 100 mM sialic acid or sterile PBS at indicated time points (arrowhead) during DSS (3%, w/v) challenge for 5 days. Relative change in body weight was monitored daily, N=8, not significant (Two-tailed Student's t-test).

Supplementary Methods

Sialidase specificity assay

The linkage specificity of caecum sialidase was determined by incubating 0.1 ml of 100 mM 3SL, 100 mM 6SL with (15%, v/v) sterile caecum fluid in at 37°C for 16 h. Reaction products were separated by HPLC using a CarboPac PA200 column (Dionex) and detected by pulsed amperometry. The running conditions were 5 min of 100 mM NaOH for 5 min, followed by a linear gradient of sodium acetate from 0 to 250 mM over 40 min at a flow rate of 0.35 ml/min. The column was rinsed with 1 M sodium acetate for 5 min and equilibrated with 100 mM NaOH for 10 min. The retention times for lactose, sialic acid, 6SL, and 3SL were 4.7, 14.0, 22.0 and 22.4 min respectively. Sialic acid concentration was determined by comparison of peak area with an authentic standard solution.

Quantitative PCR of *B. vulgatus* species

The relative abundance of specific *B. vulgatus.* in fecal samples was determined by TaqMan QPCR assay. The primers (forward primer: 5'-CGGGCTTAAATTGCAGATGA-3'; reverse primer 5'-CATGCAGCACCTTCACAGAT-3'; and the TaqMan probe 5'-FAM-TGAAAGCCGTAAGCCGCAAGG-TAMRA-3') were used as described previously[1]. Cycling condition was one cycle at 50°C for 2min, 95°C for 5min followed by 40 cycles at 95°C for 10 s and 63°C for 50 s.

Sialidase activity assay

The recombinant *B. vulgatus*_4143 sialidase was expressed and purified as previously described. The fluorogenic substrate (4-MU-NeuNAc; Carbosynth) was used to determine sialidase activity. In brief, recombinant sialidase (ca. 0.25 µg) was incubated with 0.1 mM 4-MU-NeuNAc in 0.2 ml of 100 mM sodium acetate buffer (pH 7.4) at 37°C for 15 min. Assay was stopped by adding 0.8 ml of 0.5 M sodium carbonate buffer (pH 10.5) and diluted

20-fold prior to fluorescence measurement at an excitation wavelength of 360 nm and an emission wavelength of 440 nm. 2-O-(4-Nitrophenyl)-α-D-N-acetylneuraminic acid (pNP-NeuNAc; Carbosynth) was also used to determine the sialidase activity. Recombinant sialidase was incubated with 200 µM pNP-NeuNAc in 0.2 ml of 100 mM Tris-Cl buffer (pH 7.4) at 37°C for 2 h. Assay was stopped by adding 0.2 ml of 0.4 M NaOH-glycine buffer (pH 10.8) and measured the absorption at 405nm with a microplate reader.

Reference

1. Tong, J., Liu, C., Summanen, P., Xu, H. & Finegold, S.M. Application of quantitative real-time PCR for rapid identification of Bacteroides fragilis group and related organisms in human wound samples. *Anaerobe* **17**, 64-68 (2011).
